# Psoriasis Patients' Knowledge about the Disease and Treatments

**DOI:** 10.1155/2013/921737

**Published:** 2013-06-24

**Authors:** Astrid Klopstad Wahl, Torbjørn Moum, Hilde Stendal Robinson, Eva Langeland, Marie Hamilton Larsen, Anne Lene Krogstad

**Affiliations:** ^1^Department of Health Sciences, Institute of Health and Society, University of Oslo, P.O. Box 1089, Blindern, 0317 Oslo, Norway; ^2^Institute of Basic Medical Sciences, Medical Faculty, University of Oslo, P.O. Box 1111, Blindern, 0317 Oslo, Norway; ^3^Faculty of Health and Social Sciences, Bergen University College, P.O. Box 7030, 5020 Bergen, Norway; ^4^Section for Climate Therapy, Oslo University Hospital, P.O. Box 4950, Nydalen, 0424 Oslo, Norway; ^5^Department of Dermatology, Oslo University Hospital, P.O. Box 4950, Nydalen, 0424 Oslo, Norway

## Abstract

Patients' knowledge about psoriasis and its treatment has been randomly studied previously. The aim of the study is to investigate patients' knowledge about psoriasis in relation to undergoing patient education in the context of climate therapy (CT). The psoriasis knowledge questionnaire (PKQ) was used in a follow-up pre–post study design of Norwegian patients with psoriasis at the age of 20 years and older undergoing CT at Gran Canaria (Spain). Patients completed the PKQ and provided selected demographic, clinical and health information before (T1), immediately after (T2), and 3 months after (T3) CT. Disease severity was assessed using the psoriasis area and severity index (PASI). 254 psoriasis patients were included (74%). The PKQ score improved significantly from T1 to T2 and T3 (*P* < 0.001 for both comparisons). Although patient's knowledge improved, further research should use gold standard designs (experiments) to study the effects of educational interventions in different contexts.

## 1. Introduction

Knowledge about a disease and its treatment alone does not change the attitudes and health behaviour related to living with chronic illness. Nevertheless, knowledge about a disease and its treatment is important for coping with the symptoms and living well with a chronic illness. Integration of knowledge, skills, and values enhances an individual's health competence, which in turn may affect the patient's involvement with their disease, health, work, coping, and quality of life [1]. To our knowledge, few studies have been published on psoriasis patients' knowledge about the disease, in particular in the past few decades.

Research has shown that living with psoriasis can be difficult and can affect different aspects of quality of life [2, 3]. Poor adherence to prescribed treatment is also a major problem in this patients group [4, 5]. It is reasonable to think that focusing on knowledge about the pathogenesis and treatment of psoriasis may increase the patient's perception of control and attention to aggravating factors and may thus increase patient's compliance with treatment and positive lifestyle habits. Lanigan and Layton [6] used a 22-item knowledge questionnaire developed by Lanigan and Farber [7] to assess the level of knowledge and information sources used by patients with psoriasis. Although the patients knew many core facts about psoriasis, they did not know a number of facts about the disease, which may be valuable in improving self-management. Renzi et al. [8] studied the levels of participation, satisfaction, and knowledge among patients with cutaneous psoriasis and psoriatic arthritis. The knowledge questions were related to treatment and were assessed by 12 correct and incorrect statements. According to Renzi et al. [8], the level of knowledge was not high, a finding that is consistent with previous studies [9, 10]. Insufficient patient knowledge can represent a barrier to participation in shared decision making [10].

Climate therapy (CT) at Gran Canaria combines treatments with sunlight and salt water to relieve symptoms and is a treatment option for psoriasis patients in Norway [11]. The treatment programme includes a systematic teaching module to improve knowledge about psoriasis, lifestyle choices, management of symptoms, and the impact of the disease. This programme is based on the recognition that a patient's health competence is important for their involvement in the disease, coping, quality of life, and use of health care resources. A previous study has shown positive changes in disease severity and health-related quality of life after CT, although these positive changes last only for about 2 months [12]. Few studies have investigated the effect of educational interventions on psoriasis in general and specifically with knowledge as an outcome. However, positive effects on patients' knowledge have been reported [13, 14].

Although a patient's knowledge about a disease and its treatment is not the only factor explaining success with self-management, it should not be overlooked in the modern health care system, which relies on principles of shared decision making and partnerships between doctors and patients. Although patients' knowledge about their disease and treatment has been studied previously, the assessment tools are old [6] or limited in scope; for instance, most focus only on treatment [8] or were developed for subgroups such as patients with psoriatic arthritis [15]. The overall objective of this study was to evaluate the patients knowledge of psoriasis in depth by investigating changes in knowledge in broad areas related to patient education in the context of CT. The specific aims were to

- describe the level of knowledge in a sample of patients with psoriasis entering a CT programme,
- investigate the associations between selected demographic, clinical factors and knowledge about psoriasis,
- evaluate changes in knowledge in a sample of patients with psoriasis undergoing CT (including a standard programme of patient education).

## 2. Materials and Methods

### 2.1. Study Design and Population

The present study is a follow-up, pre-post study design of Norwegian patients with psoriasis at the age of 20 years and older who were offered a 3-week CT programme at Gran Canaria. The design included three measurements over a period of 4 months. The patients were recruited to the study when they arrived at the treatment centre. They completed the questionnaire just before (T1) and immediately after CT (T2) and again 3 months after CT (T3). The study lasted from late April 2009 to early January 2010, and during this time 343 psoriasis patients completed the CT programme and were eligible for the study. Of these 343 patients, 254 (74%) agreed to be included in the study. Reasons for not willing to participate were not evaluated. For the PKQ and the purpose of this paper, the data were available for 253 (T1), 249 (T2), and 211 (T3) patients, resulting in response rates of 98% (249/254) for T2 and 83% (211/254) for T3.

The study was approved by the hospital administration and Norwegian Social Science Data Service and was recommended by the Regional Committee for Medical Research Ethics for Southern Norway. The protocol complied with the Declaration of Helsinki.

### 2.2. Climate Therapy (CT)

The 3-week CT programme comprised both a sun and sea treatment and patient education. Patients received on average 80 h of sunshine during their stay. The sun exposure was scheduled individually with respect to skin type and ultraviolet index. They were encouraged to swim frequently in salt water and use moisturizing creams. A Nordic medical team (dermatologist, nurses, and physiotherapist) monitored the patients. Patients received both individual and group-based education, guidance, and daily training. Some parts were mandatory and some parts were voluntary. The teaching programme included information and dialogue about psoriasis pathogenesis, manifestations, comorbidity, quality of life and treatment options, physical activity, and dietary aspects. Patients also participated in group discussions focusing on living with psoriasis. 

### 2.3. Instruments

#### 2.3.1. Knowledge

The PKQ, which is a simple additive index, was developed in the Norwegian dermatology context and contains 49 statements about psoriasis. The items are shown in Table 1. The response alternatives to each statement are “valid,” “uncertain,” or “invalid.” A total score is calculated by counting the number of correct answers with a possible range of 0–49. A higher score indicates greater knowledge. The development of the PKQ started with discussions among the health care personnel (nurses and doctors) at a dermatology ward about the expectations of what individuals with psoriasis should know about their disease. The first author (AKW) searched the literature (books and articles) on patient information related to psoriasis. A first draft of the questionnaire was developed from the discussions and the literature review; this draft included 56 statements and five response alternatives from 1 “agree” to 5 “disagree.” This draft was presented to health care personnel, researchers within the field, and patients. A pilot sample of 50 patients attending CT completed and commented on the questionnaire through a brief interview with ALK. After the pilot study and several rounds of discussions and comments about the statements, wording, and responses, a final version of 49 statements with the three response alternatives, valid, uncertain, and invalid was agreed upon. Cronbach's alpha for the 49 items index was 0.84 in the present sample.

In addition to the PKQ, at T2, a single item asked the patients to rate to what extent the CT programme had contributed to their knowledge about psoriasis. The possible responses were “considerably,” “some,” “little,” and “not at all.” They were also asked to answer an open-ended question about the reason(s) for their response.

#### 2.3.2. Demographic and Clinical Variables

The questionnaire contained questions about age, sex, marital status, level of formal education, cohabitation, work, duration of psoriasis, previous CT, and comorbidities. Disease severity was assessed using the psoriasis area and severity index (PASI). The diagnosis of psoriatic arthritis was confirmed by patient records.

### 2.4. Statistics

The SPSS PC version 19.0 was used to analyse the data. Descriptive analyses were performed to assess the frequency, mean, standard deviation (SD), and range of scores (minimum to maximum). Cronbach's alpha was used to estimate the internal consistency of the scale (reliability). Paired-sample *t*-tests were used to evaluate changes in the PKQ score from T1 to T2 and T3. Multiple linear regression (forward) analyses were used to identify significant associations between demographic and clinical factors (independent variables) and the PKQ score (dependent variable) at T1. *P* values < 0.05 were considered significant.

## 3. Results

### 3.1. Study Population

The mean age for the sample was 47 years (range 20–80) and comprised 40% women. Sixty percent of the sample reported <12 years of education (primary school level), and 69% were in paid work. Forty-four percent reported comorbidity, and the mean duration of psoriasis was 24 years (SD = 13). Further information about the demographic and clinical characteristics is shown in Table 2.

### 3.2. The PKQ—Descriptive at T1

The percentage of correct answers at T1 (before CT) varied greatly between the 49 PKQ items (see Table 1). The statement receiving most of the number of correct responses (95%) was “Stress and worry can/may exacerbate psoriasis” (item 14). The lowest percentage of correct answers (7%) was for the statement “Diet is important to the development of psoriasis” (item 16). Six other items had >80% correct answers: “The ways psoriasis develops vary greatly” (item 2); “People with psoriasis can develop psoriatic arthritis” (item 3); “With psoriasis, there is increased division of cells in the skin” (item 4), “If all signs of psoriasis are gone, you are completely cured” (item 5); “There are several types of cortisone ointments/creams with different potency in the treatment of psoriasis” (item 22); and “Salicylic acid ointment/oil is used to remove psoriasis scales of the skin and the scalp” (item 39). In addition to item 16, eight items were answered correctly by <20% of the participants: “Taking antibiotics can/may cause psoriasis” (item 11); “Environmental pollution can/may cause psoriasis” (item 12); “Antimalaria and high blood pressure medication can/may worsen psoriasis” (item 13); “Laundry powder and various types of chemicals can/may exacerbate psoriasis” (item 17); “Vitamin D preparations should be applied abundantly to the lesions, but the amount used should not exceed 100 grams per week” (item 25); “Vitamin A preparations (e.g., Neotigason) cannot be used during pregnancy” (item 33); and “Cyclosporin (Sandimmun Neoral) affects the immune system and can be used to treat serious cases of psoriasis” (item 36). For other statements, 50–60% of the answers were correct.

On the single question asking the patients at T2 to what extent the CT programme had improved their knowledge about psoriasis, 62% reported considerably, 34% to some extent, 3% little, and 1% not at all. When responding to the open-ended question about the reason for improvement, participants mentioned frequently the education programme and meeting other patients.

### 3.3. Association between PKQ and Demographic and Clinical Factors at T1

Results from the multiple linear regression (forward) analysis show that sex (women), higher educational level, higher PASI score, and previous CT were significantly associated with greater psoriasis-related knowledge at T1 (before CT). This model explained 20% of the variance in the PKQ score at T1. Further information is presented in Table 3.

### 3.4. Immediate and Long-Term Changes in PKQ Score following CT

The mean PKQ scores at the different measurement times (T1–T3) are shown in Table 3. The scores were 24.4 (SD 7.1), 29.7 (SD 7.0), and 29.3 (SD 7.1) at T1, T2 and T3, respectively. Paired-sample *t*-tests showed significant improvements in the PKQ score (*P* < 0.001) from T1 (before CT) to T2 (followup immediately after CT) and from T1 to T3 (followup 3 months after CT). 

Twelve percent of the patients had a lower PKQ score at T2 compared with T1, and 14% of the patients had a lower score at T3 than at T1. The PKQ score improved by 9 points in 25% of the patients from T1 to T2 and in 19% from T1 to T3.

## 4. Discussion

The present study focused on psoriasis patients' knowledge of broad medical domains. The patients completed the PKQ in the context of CT. The importance of patient knowledge in the modern health care setting is based on the philosophy of a partnership between the patient and doctor for shared decision making about treatment options. In the study by Renzi et al. [10], patients with good knowledge more frequently reported complete satisfaction with care compared with patients with poor knowledge. 

We found large variations in knowledge between items on the PKQ, which may be interpreted as favourable with regard to the questionnaire's ability to reveal strengths and weaknesses within a patient's knowledge. The statement that attracted the highest percentage of correct answers was that stress and worry can exacerbate psoriasis (item 14), whereas the statement with the lowest percentage of correct answers was the statement that diet is important to the development of psoriasis (item 16). Because knowledge profiles have been studied rarely in relation to psoriasis, there are few studies with which to compare our results. A study by Lanigan and Layton [6] in 1991 found that a number of facts about the disease were not known by patients but would be of value in self-care. For example, 53% of patients did not know that sunburn can exacerbate psoriasis, and 56% were unaware that infections can aggravate the condition. A recent study by Renzi et al. [8] found gaps in knowledge about treatments in both psoriasis and psoriatic arthritis groups. The same pattern has been reported in other studies [15, 16]. Our study also identified gaps of knowledge. We believe it is important to educate patients about their condition; as new knowledge about psoriasis develops, the nature of the knowledge patients need must be revised and refined.

The knowledge level improved significantly from before to immediately after CT and remained at the same level after 3 months, suggesting that the CT programme had an impact. Due to the programme content, we may anticipate some change in knowledge (education about the topics and patients talking with health personal and fellow patients during the CT). However, the magnitude of change is difficult to evaluate. Additional studies in different patient education settings for psoriasis are needed in order to decide on the clinical significance of the findings related to change in knowledge. Because of the lack of a control group, it is not possible to draw conclusions about cause and effect. However, because the patients' knowledge was measured by factual questions, one may assume that the CT programme, which included education, counseling, and support from other patients, influenced their knowledge level. This conclusion is also supported by the responses to the open-ended questions, which showed that most patients thought that the stay had improved their knowledge, specifically the importance of the courses and support from fellow patients. A study examining the effect of an empowerment-based educational intervention among psoriasis patients attending a medical spa showed improved knowledge about the disease in the experimental group, although knowledge was measured as only one dimension of empowerment [14]. Another study investigating the efficacy of a single educational intervention in patients with chronic plaque psoriasis found that the patients had better knowledge about the disease after the intervention [13]. However, knowledge was measured by four questions that asked patients to rate their perception of knowledge in general and related to causes, treatment, and biological therapies. It is possible that patients perceive their knowledge level to be better than what would be revealed by answering factual questions.

We found several significant associations between selected demographic and clinical variables and knowledge. Being a woman, living with someone, higher educational level, having more severe disease, and previous participation in CT were significantly related to better knowledge at baseline. Our findings contrast with those of Lanigan and Layton [6], Lubrano et al. [15] and Tham and Tay [16], who reported no associations between demographic and clinical variables and knowledge level in patients with psoriasis or psoriatic arthritis. However, Lubrano et al. [15], found a significant association between educational level and knowledge. It is reasonable to expect that people with a higher educational level are more interested in knowledge, and perhaps this is also the case for women. Older people and those who attended more CT may have had the disease for longer and thereby learned more about this disease with time. People with more severe disease may experience the importance of medical knowledge in order to keep symptoms under control. 

Based on the results from both the developmental stages of the PKQ and the empirical study, we consider the PKQ a proper tool for measuring knowledge in the setting of psoriasis. The instrument could be used in clinical practice to create a patient knowledge profile to individualize patient information and education. 

Further research should use gold standard designs to study the effects of different educational interventions on knowledge and to evaluate the relationships between patient knowledge, self-management skills and competence, and quality of life.

## Figures and Tables

**Table 1:** The PKQ items: percentages of correct and uncertain answers at baseline (T1).

The 49-item PKQ	True/false	Percentage of correct answers	Uncertain
(1) Once you have psoriasis, the illness will become increasingly worse.	False	41	27
(2) The ways psoriasis develops vary greatly.	True	91	7
(3) People with psoriasis can develop psoriatic arthritis.	True	91	7
(4) With psoriasis, there is increased division of the cells in the skin.	True	89	9
(5) If all signs of psoriasis are gone, you are completely cured.	False	84	14
(6) The more psoriasis you have, the greater the chances for developing psoriatic arthritis.	False	30	59
(7) Scaling of the skin is the result of accumulation of dead cells.	True	78	16
(8) With psoriasis, the skin feels warm because of increased blood flow in the psoriatic lesions.	True	31	48
(9) A throat infection can/may cause psoriasis.	True	62	27
(10) A skin injury can/may cause psoriasis.	True	71	20
(11) Taking antibiotics can/may cause psoriasis.	False	11	65
(12) Environmental pollution can/may cause psoriasis.	False	17	67
(13) Antimalaria and high blood pressure medicines can/may worsen psoriasis.	True	11	81
(14) Stress or worry can/may exacerbate psoriasis.	True	95	3
(15) Sunlight can/may exacerbate psoriasis for some people.	True	51	31
(16) Diet is important to the development of psoriasis.	False	7	36
(17) Laundry powder and various types of chemicals exacerbate psoriasis.	False	8	55
(18) Scratching the skin can/may worsen psoriasis.	True	56	31
(19) Treatment is the same for all psoriasis patients.	False	79	13
(20) Treatment depends on lifestyle, the severity of psoriasis, and the location of the lesions.	True	63	23
(21) Cortisone ointment/cream may be applied only for one week at a time.	False	36	29
(22) There are several types of cortisone ointments/creams with different potency for the treatment of psoriasis.	True	91	8
(23) Research shows that herbal medicines have a positive effect on psoriatic lesions.	False	20	71
(24) Daivonex (calcipotriol) ointment/cream contains a substance similar to vitamin D.	True	55	42
(25) Vitamin D preparations should be applied abundantly to the lesions, but the amount used should not exceed 100 grams per week.	True	15	71
(26) All topical treatments for psoriasis can/may be applied at all skin locations over the entire skin surface.	False	43	42
(27) Phototherapy can/may increase the risk of skin cancer.	True	36	47
(28) Sunlight suppresses the immune system of the skin.	True	25	39
(29) Methotrexate is used in the treatment of psoriatic lesions and arthritis.	True	65	34
(30) Moisturizers stop the skin's ability to produce fatty substances, and thereby you become dependent on using them to keep your skin moist.	False	46	46
(31) There is no upper limit regarding UVB treatment for psoriasis.	False	63	29
(32) Daivobet ointment contains only cortisone.	False	37	52
(33) Vitamin A preparations (e.g., Neotigason) cannot be used during pregnancy.	True	19	79
(34) Carbamide-containing moisturizers can/may reduce the scaling.	True	43	55
(35) Creams contain more fat than ointments.	False	43	43
(36) Cyclosporin (Sandimmun Neoral) affects the immune system and can be used to treat serious cases of psoriasis.	True	19	80
(37) Psoriasis located in nails is difficult to treat.	True	62	36
(38) Ointment is often a better treatment for psoriasis than cream.	True	52	39
(39) Salicylic acid ointment/oil is used to remove psoriasis scales of the skin and the scalp.	True	89	10
(40) The effect of locally applied cortisone may decrease with daily use over several months.	True	61	36
(41) Enbrel/Remicade heals psoriasis.	False	25	59
(42) Psoriasis is an inflammation of the skin.	True	53	25
(43) Psoriasis is an infectious disease.	False	53	36
(44) Psoriasis is a disease of the immune system.	True	43	32
(45) Psoriasis is an allergic disease.	False	67	28
(46) Women are more prone to psoriasis than men.	False	46	56
(47) Psoriasis can pass between generations.	True	77	21
(48) Psoriasis occurs most frequently in people older than 50 years.	False	51	42
(49) Overweight, diabetes, and cardiovascular diseases occur more frequently in people with psoriasis than in the general population.	True	31	60

**Table 2:** Descriptive data, *n* = 254.

	*n*	Range (mean) SD	*n* (%)
Age, years	254	20–80 (47) 12	
Years with psoriasis	245	1–60 (24) 13	
BMI	251	17–49 (28) 5	
Pretreatment PASI score	253	0.4–26.1 (7.5) 4.1	
Posttreatment PASI score	253	0–20.6 (1.6) 2.2	
Sex, women	254		102 (40)
Educational level	254		
Primary school ≥ 12 years			153 (60)
University < 4 years			52 (20)
University ≥ 4 years			49 (19)
Employed, yes	254		174 (69)
Living alone, yes	250		70 (28)
Comorbidity, yes	254		111 (44)
PsA verified by doctor, yes	252		64 (25)
Previous climate therapy, yes	252		140 (55)
PKQ T1	253	5–43 (24.4) 7.0	
PKQ T2	249	6–44 (29.7) 7.0	
PKQ T3	211	9–43 (29.3) 7.1	

BMI: body mass index, PsA: psoriasis arthritis, PASI: psoriasis area severity index, PKQ: psoriasis knowledge questionnaire, T1: before climate therapy, T2: immediately after climate therapy, and T3: three months after climate therapy.

**Table 3:** Associations between demographic and clinical factors and PKQ by simple (unadjusted) and multiple (forward) (adjusted) linear regression analysis at T1.

	Patients' level of knowledge (PKQ) (higher score indicates greater knowledge)	
	Unadjusted Stdandard beta coefficient (*P* value)	Adjusted Stdandard beta coefficient (*P* value)
Sex (1 = men; 2 = women)	0.20 (0.001)	0.25 (0.000)
Cohabitation (0 = together; 1 = alone)	ns	ns
Education (1–5: higher score = higher level of education)	0.25 (0.000)	0.27 (0.000)
Age (years)	ns	ns
BMI (higher score = higher BMI)	ns	ns
PASI (higher score = more severe disease)	ns	0.19 (0.002)
PsA (1 = no; 2 = yes)	0.14 (0.023)	ns
Comorbidity (1 = no; 2 = yes)	ns	ns
Previous climate therapy (1 = no; 2 = yes)	0.28 (0.000)	0.29 (0.000)

BMI: body mass index, PsA: psoriasis arthritis, PASI: psoriasis area severity index, PKQ: psoriasis knowledge questionnaire, T1: before climate therapy, and ns: nonsignificant.

## References

[1] Osborne RH, Elsworth GR, Whitfield K (2007). The Health Education Impact Questionnaire (heiQ): an outcomes and evaluation measure for patient education and self-management interventions for people with chronic conditions. *Patient Education and Counseling*.

[2] de Korte J, Sprangers MAG, Mombers FMC, Bos JD (2004). Quality of life in patients with psoriasis: a systematic literature review. *Journal of Investigative Dermatology Symposium Proceedings*.

[3] Sampogna F, Tabolli S, Abeni D (2012). Living with psoriasis: prevalence of shame, anger, worry, and problems in daily activities and social life. *Acta Dermato-Venereologica*.

[4] Richards HL, Fortune DG, O’Sullivan TM, Main CJ, Griffiths CEM (1999). Patients with psoriasis and their compliance with medication. *Journal of the American Academy of Dermatology*.

[5] Zaghloul SS, Goodfield MJD (2004). Objective Assessment of Compliance with Psoriasis Treatment. *Archives of Dermatology*.

[6] Lanigan SW, Layton A (1991). Level of knowledge and information sources used by patients with psoriasis. *British Journal of Dermatology*.

[7] Lanigan SW, Farber EM (1990). Patients’ knowledge of psoriasis: pilot study. *Cutis*.

[8] Renzi C, Di Pietro C, Tabolli S (2011). Participation, satisfaction and knowledge level of patients with cutaneous psoriasis or psoriatic arthritis. *Clinical and Experimental Dermatology*.

[9] Leung Y-Y, Tam LS, Lee KW, Leung MH, Kun EW, Li EK (2009). Involvement, satisfaction and unmet health care needs in patients with psoriatic arthritis. *Rheumatology*.

[10] Renzi C, Di Pietro C, Gisondi P (2006). Insufficient knowledge among psoriasis patients can represent a barrier to participation in decision-making. *Acta Dermato-Venereologica*.

[11] Norwegian Official Reports (NOU)

[12] Wahl AK, Mørk C, Cooper BA, Padilla G (2005). No long-term changes in psoriasis severity and quality of life following climate therapy. *Journal of the American Academy of Dermatology*.

[13] Lora V, Gisondi P, Calza A, Zanoni M, Girolomoni G (2009). Efficacy of a single educative intervention in patients with chronic plaque psoriasis. *Dermatology*.

[14] Pagliarello C, Calza A, Armani E, di Pietro C, Tabolli S (2011). Effectiveness of an empowerment-based intervention for psoriasis among patients attending a medical spa. *European Journal of Dermatology*.

[15] Lubrano E, Helliwell P, Parsons W, Emery P, Veale D (1998). Patient education in psoriatic arthritis: a cross sectional study on knowledge by a validated self-administered questionnaire. *Journal of Rheumatology*.

[16] Tham SN, Tay YK (1995). A questionnaire-based survey of patients’ knowledge of psoriasis at the National Skin Centre. *Annals of the Academy of Medicine Singapore*.

